# Outcome of open excisional breast biopsies in Abakaliki, South-East Nigeria

**DOI:** 10.11604/pamj.2018.31.185.15216

**Published:** 2018-11-15

**Authors:** Maradona Ehikioya Isikhuemen, Monday Osaze Eliboh, Uche Emmanuel Eni, Kenneth Chinedu Ekwedigwe, Ileogben Sunday-Adeoye

**Affiliations:** 1 Department of Obstetrics and Gynaecology, University of Benin Teaching Hospital, Benin City, Edo State, Nigeria; 2 National Obstetric Fistula Centre, Abakaliki, Ebonyi State, Nigeria; 3 Department of Surgery, Federal Teaching Hospital, Abakaliki, Ebonyi State, Nigeria

**Keywords:** Open excision breast biopsy, complications, Abakaliki, Nigeria

## Abstract

**Introduction:**

open excisional breast biopsy is a known modality for treatment of breast lumps especially in developing countries. Other sophisticated methods are available for management of breast lumps in more advanced nations. Our aim in this study was to review the outcome of open excision breast biopsies in our setting with a view to improving patient management.

**Methods:**

this study was conducted at the National Obstetric Fistula Centre, Abakaliki, South East Nigeria among women who had excision breast biopsy between January 2015 and December 2016. Data was analysed using Statistical Package for Social Sciences (SPSS), version 21.

**Results:**

a total of 107 case folders were reviewed in this study. The mean age of the women was 27 ± 10 years. Overlying breast incision was the preferred route in 78(72.9%), periareolar incision in 28(26.2%), and Gillard Thomas's method (infero-lateral submammary sulcus incision) used in one patient with bilateral multiple breast lumps (0.9%). The complications recorded in this study were haematoma in 3(2.8%), wound infection in 5(4.7%) and wound breakdown in 1(0.9%). Hypertrophic scar was found in 2(1.8%) patients at follow-up. Overall, most patients were satisfied with the aesthetic outcome of their surgery.

**Conclusion:**

open excision breast biopsy is a useful modality for management of breast diseases in our setting. Complication rates are minimal. Both overlying and periareolar breast incisions results in aesthetically satisfactory scar in our practice. Inferior-lateral sub mammary sulcus skin incision is useful when the lumps are multiple and located at different quadrants of the breast. Appropriate use of drain helps to reduce the incidence of haematoma.

## Introduction

Breast lump is a common source of worry to the affected woman. It is a common presentation to the surgical outpatient department. Although most patients with breast lump will want it removed, they are predominantly benign. Removal of breast lumps may be done using open excision biopsy method. This method is readily available in our setting. A periareolar skin incision may be used during this procedure or an incision may be made directly over the breast mass [1]. A better cosmetic outcome has been reported with the periareolar incision [1]. However, minimally invasive methods including ultrasound guided vacuum-assisted biopsy have been described for breast lump removal [2, 3]. Also Endoscopic excision of breast fibroadenoma through the axilla which spared the breast from scar has also been described [4]. These sophisticated methods are not readily available in our setting probably due to the high cost of equipment and the expertise required. The commonest type of benign breast lump is fibroadenoma. Other common types of benign breast lumps include fibrocystic change, duct ectasia with periductal mastitis, chronic non specific mastitis, sclerosing adenosis, benign phylloides, intraductal papilloma and lipomata. Less common benign breast lesions include chronic breast abscess, fat necrosis and epidermal cyst [5- 7]. Excision breast biopsies for benign breast lesions appear more common than those for malignant lesions in our setting partly because most breast malignancies present at late stages when excisional biopsy is inappropriate. One reason for this late presentation is under utilization of screening mammography in our setting [8]. Conservative management of breast lumps may be appropriate if all components of the triple assessment test are negative and if there are no cosmetic concerns on the part of the patient. Though there are laid down indications for excision breast biopsy, this may not always be applicable as some patients fear that their lumps may harbour cancer and would want the tumour removed. Excision breast lump biopsy is clearly indicated when the true nature of the mass is in doubt due to discordance in the triple assessment tests, as it offers opportunity for histological evaluation of the specimen. This allows for a definitive diagnosis and appropriate treatment of the disease. Excision biopsy of suspected malignant lesion is also indicated for small lesions not amenable to fine needle aspiration cytology (FNAC) or core biopsy. Complications can arise from any surgical procedure including open excision biopsy of breast lumps. Previously reported complications are bleeding, haematoma formation, incomplete excision and marked scaring [1, 3]. A poor cosmetic outcome is another issue which probably no young woman would be comfortable with. Our aim in this study was to review the outcome of open excision breast biopsies in our setting with a view to improving patient management.

## Methods

This retrospective study was conducted at the National Obstetric Fistula Centre, Abakaliki, South East Nigeria. The primary function of the centre is to provide free surgical services to patients with genital fistula. It also has a general surgery clinic dedicated for the management of breast diseases. Case records of 107 women who had excision biopsy between January 2015 and December 2016 were reviewed. Data was collected using a structured proforma and analysed using the Statistical Package for Social Sciences (SPSS) Version 21. Patients that had incision biopsies during the study period were excluded. Ethical clearance was gotten to review case folders from the Ethics and Research Committee of the National Obstetric Fistula Centre, Abakaliki. After a clinical examination, all patients had fine needle aspiration cytology. They were subsequently counseled following the FNAC result and those that gave consent for lump removal had free open excision breast biopsy. All were done as day cases. However, patients with large breast lumps and who had wound drain inserted were post-operatively observed on the ward till drain removal. All but one case were done by local anaesthesia; one patient with multiple bilateral breast lumps was done under general anaesthesia using inferior-lateral sub mammary sulci incisions to harvest all the lumps in both breasts. Haemostasis was by suture ligation, as the centre had no diathermy presently. Wound closure was by interrupted subcutaneous suture with buried knot using absorbable vicryl 2/0 strands. This leaves the patient with no suture for removal. We gave our patients prophylactic antibiotics and oral analgesics. Specimens were then sent for histology. Patients were followed up after surgery and were discharged from clinic only after the histology result confirmed as benign lesion. Those with malignant histology were treated as appropriate. Patients that had any complications were managed as necessary.

## Results

The mean age of the patients was 27 ± 10 years. Their age ranged from 14 to 60 years. Most (97.2%) were Igbo and 67 (62.6%) were students. The sociodemographic indices were shown in Table 1. Their parity ranged from 0 to 11 with a mean parity of 1 ± 2. Symptom duration was from 1 to 180 months with a mean duration of 17 months. Size of breast lump ranged from 0.5-16cm in the widest diameter (mean diameter was 4cm). Interval between first presentation and surgery was between 1 and 72 weeks (mean 7 weeks). Number of clinic visit before surgery was between 1 and 6 (mean 2). Duration of follow up after surgery was from 1 to 79 weeks. The mean duration of follow up was for 3 weeks. Number of follow up visit was between 1 and 6 (mean = 2). A total of 49 (45.8%) had right breast lump while 50 (46.7) had left breast lump. Bilateral breast lumps were seen in 8 (7.5%) of patients. The upper lateral region was the most common site for breast lump as seen in 37 (34.6%) of patients. Breast pain at presentation was present in 27 (25.2%) of patients. The most common clinical diagnosis was fibroadenoma (Table 2). Fibroadenoma was the most commonly excised breast mass as seen in 78(72.9%) of patients, followed by fibroadenosis in 13 (12.1%). Four suspected cases of early breast carcinoma were positive for malignant cells at FNAC and confirmed to be invasive ductal carcinoma at histology, constituting 3.8% (Table 3). Overlying incision was used for breast lump excision in 78 (72.9%) of patients while periareolar incision was used in 28 (26.2%) of patients; bilateral inferior-lateral mammary sulci incisions was used in 1(0.9%). All were done as day cases. However, 11 (10.4%) patients had drain inserted and were admitted till drain removal, not later than three days in all the patients. At the first clinic visit which was one week post operation, 83 (77.6%) had no problems, 11 (10.3%) had significant pain at operation site requiring further analgesics, 5 (4.7%) had wound infection, 3(2.8%) had hematoma and 1(0.9%) had wound break down. This is shown in Table 4. All were treated successfully with regular wound dressing and oral antibiotics on outpatient basis. Two patients were noted to develop broad heaped up (hypertrophic) scars during follow up, and were treated satisfactorily with intra-lesional triamcinolone injections. Recurrent breast lump was found in 1 (0.9%) patient. Only 3 (2.8%) requested for sick off after surgery. Hypertension as seen in 3(2.8%) of the patients was the only co-morbidity that was recorded.

**Table 1:** socio-demographic variables of the study group

Variable	Frequency (%)
**Age**	
10 – 19	26 (24.3)
20 – 29	50 (46.7)
30 – 39	13 (12.1)
40 – 49	13 (12.1)
50 – 59	4 (3.7)
60 – 69	1 (0.9)
**Tribe**	
Igbo	104 (97.2)
Hausa	1 (0.9)
Efik	1 (0.9)
Ijaw	1 (0.9)
**Occupation**	
Student	67 (62.6)
Farming	6 (5.6)
Trading	10 (9.3)
Public Servant	15 (14)
Artisan	9 (8.1)
**Level of Education**	
Primary	8 (7.5)
Secondary	60 (56.1)
Tertiary	34 (31.8)
No Formal Education	5 (4.7)
**Marital Status**	
Married	31 (29)
Single	75 (70.1)
Widow	1 (0.9)

**Table 2:** clinical diagnosis of patients

Clinical diagnosis	Frequency (%)
Early mitotic lesion	7 (6.5)
Fibroadenoma	80 (74.8)
Fibroadenosis	8 (7.5)
Duct ectasia	3 (2.8)
Lipoma	4 (3.7)
Galactocoele	2(1.9)
Phylloides tumour	1(0.9)
Paget’s disease	1(0.9)
Epidemoid cyst	1(0.9)

**Table 3:** FNAC diagnosis of patients

FNAC diagnosis	Frequency (%)
Breast cancer	1 (0.9)
Suspicious for malignancy	3 (2.8)
Fibro adenoma	78 (72.9)
Fibro adenosis	13 (12.1)
Duct ectasia	2 (1.9)
Lipoma	6(5.6)
Galactocoele	2(1.9)
Reactive node	1(0.9)
Hypertrophic scar	1 (0.9)

**Table 4:** problems at first follow up visit

Problem	Frequency (%)
Wound breakdown	1 (0.9)
Pain at operation site	11 (10.3)
Haematoma	3 (2.8)
Wound infection	5 (4.7)

## Discussion

Excision breast biopsy has continued to be an important aspect in the management of benign breast lesions. As shown in this study, majority of patients had excisional biopsy as a result of breast fibroadenoma. Fibroadenoma constitutes the majority of benign breast lumps as seen in previous studies [5, 6]. The age range of patients that had surgery in this study is similar to the age range of patients reported to have benign breast lesions in a previous survey [8, 9]. These findings are not unexpected because the incidence of breast malignancy tends to increase with age. Also excisional biopsy was predominantly for benign breast lesions because it is not usually the treatment of choice for malignant lesions in our setting where most cases of breast cancer would present at late stages. Most of the patients opt for excisional biopsy of their breast lumps out of cosmetic concerns and others out of anxiety for fear of harbouring malignancy. This was despite reassurance following negative triple assessment. The complications observed from this study were mainly wound infection and haematoma formation in a few patients. Haematoma occurred in patients with large lumps greater than 5cm, but who had no wound drain inserted. On the other hand, none of the patients with similarly large lumps who had wound drain inserted developed haematoma. Therefore, the use of wound drain when appropriate during breast excision biopsy may help reduce this complication. This is supported by other authors [10]. The infection rate of 4.7% is within the low range commonly quoted for clean surgeries [11]. However, prophylactic antibiotics are not usually given for clean surgeries. We gave prophylactic antibiotics to our patients as there were operated as day cases and they are mostly rural dwellers who may not maintain good hygiene. The role of antibiotics in achieving low infection rate in this study is not certain and can only be verified by a randomized prospective study. Wound break down occurred in one patient that had excision of diffuse duct ectasia. Periductal mastitis is known to complicate long standing duct ectasia with potential infection. This could account for the wound failure in that one case resulting in wound breakdown. Two patients had hypertrophic scars. Formation of hypertrophic scars may not be related to the surgeon's skills as it is known to naturally occur in some people following even minor injuries.

In this study, periareolar and overlying incisions were used. Patients that had overlying skin incision did not report dissatisfaction with the incision scar at follow up. A previous study reported a better cosmetic outcome with periareolar incision [1]. The periareolar incision is usually not clearly visible due to the dark areolar area. Complication rate was similar irrespective of the type of incision used. The skill of the surgeon and the deliberate use of small incisions where possible may have accounted for the good cosmetic outcome for both types of incision and the low complication rate. Our practice of wound closure by interrupted subcutaneous method with absorbable vicryl suture leaves the patient with no suture marks on the skin. This has been reported to have better aesthetic outcome [12]. As shown in this study, a good number of patients were lost to follow up. This occurred despite counseling done before surgery that the definitive diagnosis will be made on histology. Patients may not want to attend clinic after surgery because they feel that treatment of their problem ends with excision biopsy. This is usually a problem in our setting. Another reason why patients were lost to follow up may be because of the cost of transportation. Patients presented in this study were offered free investigations and treatment which encouraged them to come from far places. After surgery such patients may not adhere to follow up visit due to the distance between their homes and the hospital. A few patients in this study required a wound drain. This was reserved for patients with large breast lumps. Being a low resource setting, the tubing of infusion giving sets were fenestrated and adapted as tube drain when required. None of our patients with wound drain suffered any postoperative complication. The outcomes of excision breast biopsies were generally good. Majority of patients were able to return to work most times without requesting for sick leave. Even those that needed breast drain were fit for work on the third postoperative day after removal of drain. This would mean that breast excision biopsies may not constitute so much of economic burden. In this study, a good number of patients had surgery without delay. This is of importance to us in terms of the cost incurred by the patient when there are multiple clinic visits. The first visit is basically to investigate the patient. With the availability of the results at the second clinic visit, the patient is then planned for surgery. In our setting this may improve patient’s compliance as they are usually not happy with multiple clinic visit as a result of both direct and indirect cost in terms of time lost from work and distance to the hospital.

## Conclusion

Excision biopsy remains a useful modality for treatment of breast lumps as it allows definitive diagnosis to be made on histology. Complications from this procedure are minimal. Both periarolar and overlying breast incisions have satisfactory outcomes and are acceptable to patients. Follow up is essential following excision breast biopsy so that a histological diagnosis can be made. Recovery in the postoperative period following excision breast biopsy is excellent.

### What is known about this topic

- Excisional breast biopsy is useful in patients with breast lump.

### What this study adds

- In our setting use of excisional breast biopsy appears to be common with less complications and good healing irrespective of the type of incision. Despite the unavailability of modern instruments for breast surgery, outcome is good in our setting.

## Competing interests

The authors declare no competing interests.
